# Supplementary material for: Empirical methods for controlling false positives and estimating confidence in ChIP-Seq peaks
Source: BMC Bioinformatics. 2008 Dec 5;9:523. doi: 10.1186/1471-2105-9-523 (PMC2628906; doi:10.1186/1471-2105-9-523)
Supplement: Additional File 1 — A variety of html documents from the USeq web site detailing the available applications, their best usage, output file type descriptions, command line menus, etc. [file 1471-2105-9-523-S1.zip › USeqUserGuides/JavaDoc.zip!/JavaDoc/selex/package-summary.html]

selex


|  |  |  |
| --- | --- | --- |
| **Overview** \| **Package** \| Class \| **Use** \| **Tree** \| **Deprecated** \| **Index** \| **Help** | |  |
| **PREV PACKAGE**   **NEXT PACKAGE** | **FRAMES**    **NO FRAMES**     **All Classes** |  |


---

## Package selex

For processing of raw SELEX sequence data.

**See:**
  
          **Description**

| **Class Summary** | |
| --- | --- |
| **SelexParams** | Hold parameters and results generated by the `SelexSeqParser`; also generates reports. |
| **SelexSeqParser** | Parses out oligos from multiple rounds of SELEX. |
| **SeqFiles** | Continer for raw sequence information, seqFile, qualFile, and the actual reads from each. |
| **SeqRead** | Container for information related to a particular sequencing read. |
| **SubSeq** | Holds information related to a particular parsed SELEX oligo. |
| **UtilSelex** | Utilites for the Selex package. |

## Package selex Description

For processing of raw SELEX sequence data.

---


|  |  |  |
| --- | --- | --- |
| **Overview** \| **Package** \| Class \| **Use** \| **Tree** \| **Deprecated** \| **Index** \| **Help** | |  |
| **PREV PACKAGE**   **NEXT PACKAGE** | **FRAMES**    **NO FRAMES**     **All Classes** |  |


---
